# Biomarker discovery and metabolic profiling in serum of cardiovascular disease patients with untargeted metabolomics and machine learning

**DOI:** 10.1002/ctm2.1722

**Published:** 2024-06-20

**Authors:** Xia Shen, Shuyuan Guo, Ningning Liang, Mingming Zhao, Chun Wang, Zi Li, Dewen Yan, Lemin Zheng, Huiyong Yin

**Affiliations:** ^1^ CAS Key Laboratory of Nutrition, Metabolism, and Food Safety Shanghai Institute of Nutrition and Health Chinese Academy of Sciences (CAS) Shanghai China; ^2^ University of Chinese Academy of Sciences Beijing China; ^3^ School of Life Science and Technology ShanghaiTech University Shanghai China; ^4^ Department of Biomedical Sciences The Tung Biomedical Sciences Centre Jockey Club College of Veterinary Medicine and Life Sciences State Key Laboratory of Marine Pollution (SKLMP) The Shenzhen Research Institute and Futian Research Institute City University of Hong Kong Hong Kong China; ^5^ School of Basic Medical Sciences The Institute of Cardiovascular Sciences State Key Laboratory of Vascular Homeostasis and Remodeling NHC Key Laboratory of Cardiovascular Molecular Biology and Regulatory Peptides Beijing Key Laboratory of Cardiovascular Receptors Research Health Science Center Peking University Beijing China; ^6^ Department of Endocrinology Shenzhen Second People's Hospital the First Affiliated Hospital of Shenzhen University Health Science Center of Shenzhen University Shenzhen Clinical Research Center for Metabolic Diseases Shenzhen Center for Diabetes Control and Prevention Shenzhen Guangdong China; ^7^ Beijing Tiantan Hospital China National Clinical Research Center for Neurological Diseases Advanced Innovation Center for Human Brain Protection The Capital Medical University Beijing China; ^8^ School of Basic Medical Sciences The Institute of Systems Biomedicine Health Science Center Peking University Beijing China

Dear Editor,

Here, we employed a nontargeted metabolomics to examine serum metabolic profiles in a Chinese cohort of 243 patients with coronary heart disease (CHD) and myocardial infarction (MI), and identified 48 and 46 differential metabolites to distinguish CHD and MI from control, respectively. Employing statistical and Least Absolute Shrinkage and Selection Operator (LASSO) methodology, we built a model based on three polar metabolites, arginine, hypoxanthine and acetylcarnitine, to discriminate CHD from MI with an area under the curve (AUC) of .92 and .88 in the training and test set, respectively.

Metabolomics emerges as an enabling technique to identify circulating metabolites as potential disease biomarkers, including cardiovascular diseases (CVD).^1^ Although dysregulation of lipid metabolism has been implicated in CVD, yet a comprehensive analysis of their underlying metabolomic profiles and disease‐specific metabolic biomarkers is lacking, especially for polar metabolites.^2^ In a large cohort of 10741samples, Zeller et al. found five phosphatidylcholines (PCs) were negatively correlated with CHD,^3^ while Wittenbecher et al. found a significant correlation of Ceramide 16:0 and PC 32:0 in heart failure.^4^ Furthermore, combining metabolomics with machine learning algorithms holds enormous promise to build better diagnostic models for various human diseases.^5^, ^6^

In this study, we collected serum samples from 73 MI patients, 83 CHD patients and 87 controls and identified a total of 702 metabolites using nontargeted metabolomics (Table 1, Figure 1). Principal component analysis (PCA) was conducted to evaluate the intrinsic metabolic variations and data quality. As shown in Figure 2A, the tight clustering of quality control (QC) samples indicated good reproducibility of our metabolomic data. The MI patients showed a better separation from the control than CHD. This observation suggests smaller metabolic alterations in patients with CHD relative to MI and control groups. To gain a further insight into the metabolomic profiles among three groups, we performed a supervised partial least‐squares discriminant analysis (PLS‐DA)^7^ (Figure 2B) and found that the MI group showed good separation from the CHD and control groups. The CHD group also showed better separation from the control groups, and the *Q* ^2^ and *R* ^2^ of PLS‐DA is .671 and .857, respectively, indicative of robust performance.

**TABLE 1:** Clinical characteristics of the human subjects in this study.

	Control (*n* = 87)	CHD (*n* = 83)	MI (*n* = 73)
Gender Male (%)	64 (73.6%)	63 (75.9%)	55 (75.3%)
Age (mean (SD))	61.00 (7.90)	63.37 (10.07)	63.08 (9.30)
BMI (mean (SD))	26.41 (4.89)	25.63 (2.87)	24.51 (2.67) ^*^, ^#^
TG (mean (SD))	1.56 (0.87)	1.82 (1.52)	1.90 (4.48)
HDL (mean (SD))	1.14 (0.27)	1.13 (0.29)	1.09 (0.25)
LDL‐C (mean (SD))	2.23 (0.79)	2.53 (0.96)^*^	2.52 (0.97)^*^
apoA1 (mean (SD))	1.29 (0.27)	1.30 (0.23)	1.21 (0.20)^*^, ^#^
apoB (mean (SD))	0.75 (0.23)	0.87 (0.29)^*^	0.83 (0.26)^*^

*Note*: Data are *n*/*N* (%) or mean (SD).

Abbreviations: apoA1, Apolipoprotein A1; apoB, Apolipoprotein B; BMI, body mass index; HDL, high‐density lipoproteins; LDL, low‐density lipoproteins; TG, triacylglyerides.

* *p* < .05, compared to control.

^#^ *p* < .05, compared to the CHD group; Student's *t*‐test and Fisher's exact test were used.

**FIGURE 1:**
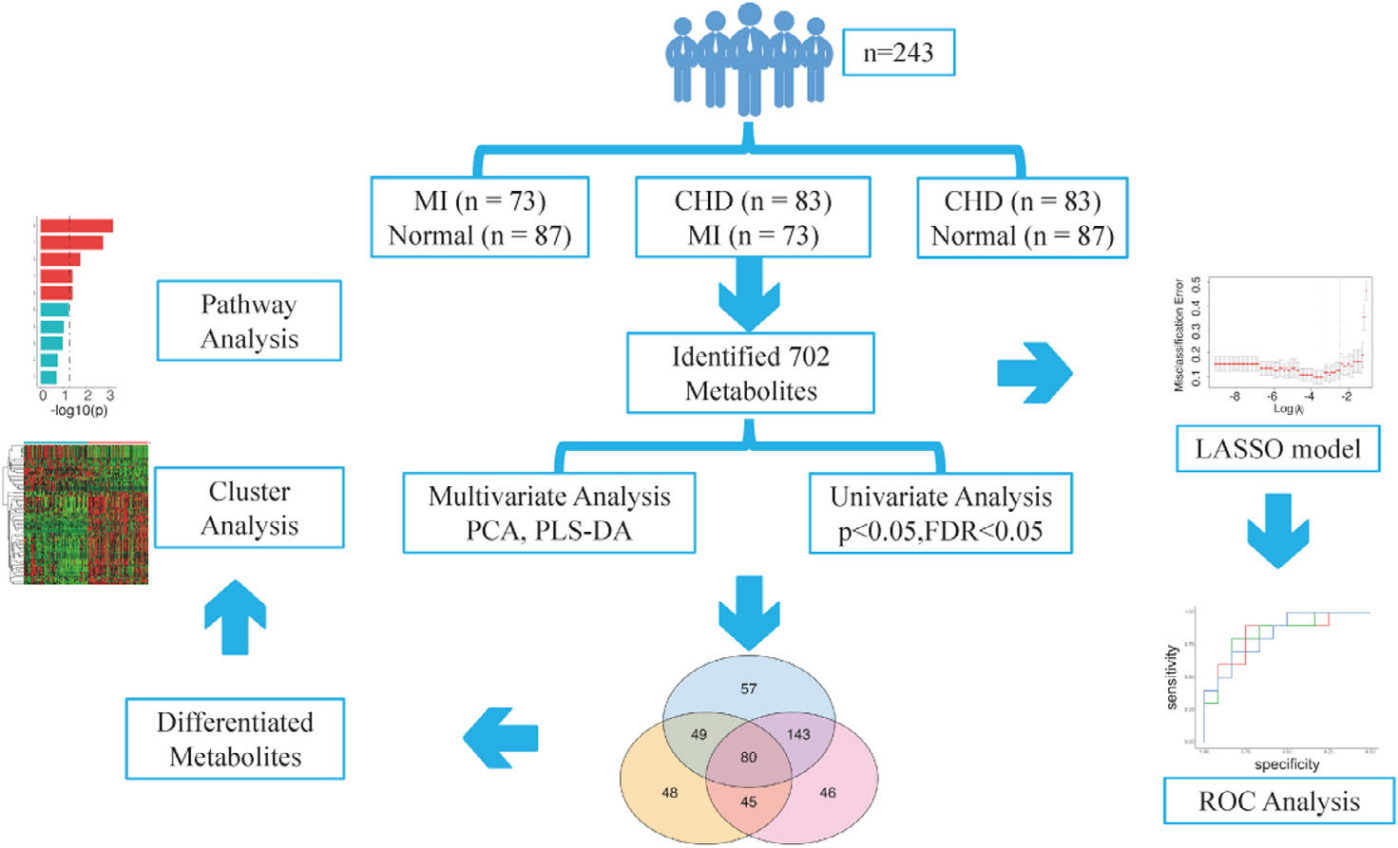
Study workflow.

**FIGURE 2:**
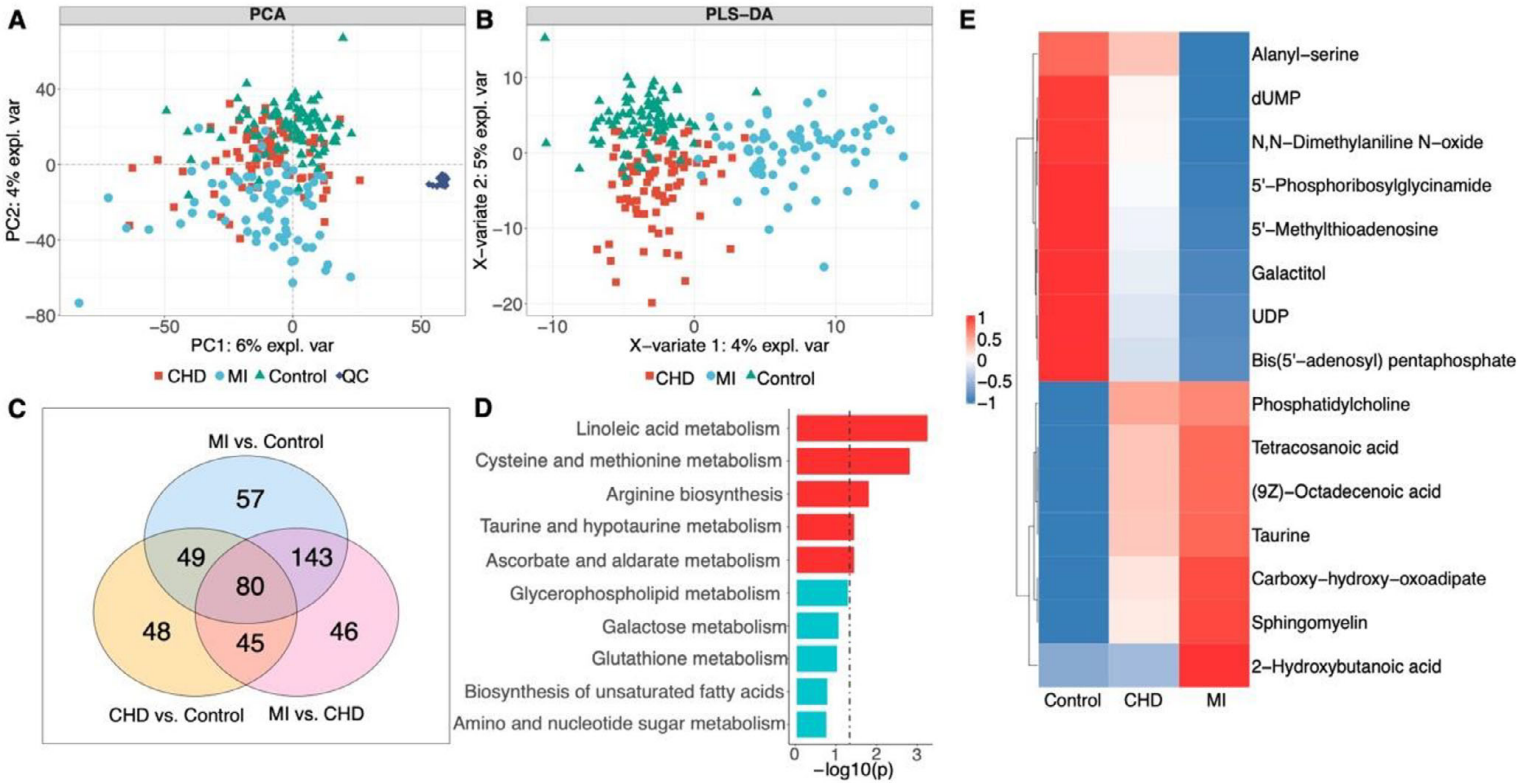
Metabolic profiles overview. (A) PCA score plot of all samples (*n* = 243). (B) PLS‐DA plots of three groups (*n* = 243). (C) Venn diagram of differentiated metabolites in MI (*n* = 73), CHD (*n* = 83) and Control (*n* = 87). (D) KEGG pathway enrichment analysis conducted on 80 metabolites with significant differences across three groups (Hypergeometric test). The pathway enrichment analysis was conducted utilising MetaboAnalyst. (E) Heatmap depicting the 15 metabolites exhibiting a unidirectional changing trend among the 80 metabolites. *p* Values were calculated by Wilcoxon test.

We next performed a univariate analysis to define significantly differentiated metabolites between three groups (FDR < .05). To this end, 80 metabolites were significantly dysregulated among three groups (Figure 2C). We subjected these 80 metabolites to Kyoto Encyclopedia of Genes and Genomes (KEGG) pathway enrichment analysis to explore the metabolic pathways dysregulated during CHD and MI progression. We observed five significantly dysregulated metabolic pathways (Figure 2D). Among the 80 differentiated metabolites, we found 15 metabolites with unidirectional trend during the disease progression trajectory, from healthy control, to CHD and MI (Figure 2E).

After the systematic analysis of the metabolic profiles among three groups, we selected the metabolites that specifically altered in patients with CHD as compared with the control group. We filtered 48 metabolites that were significantly dysregulated (Figure 2C), and details of the upregulated and downregulated metabolites are shown in Figure 3A. Overall, 37 upregulated and 11 downregulated metabolites were subjected to KEGG pathway enrichment analysis, and alpha‐linolenic acid metabolism was the significantly dysregulated pathway in CHD patients (Figure 3B). Next, we identified 57 metabolites specifically dysregulated in MI patients (Figure 2C) with 30 upregulated and 27 downregulated metabolites (Figure 3C). KEGG pathway analysis found three significantly dysregulated pathways: fructose and mannose metabolism, glycolysis/gluconeogenesis, and alanine, aspartate and glutamate metabolism pathways (Figure 3D).

**FIGURE 3:**
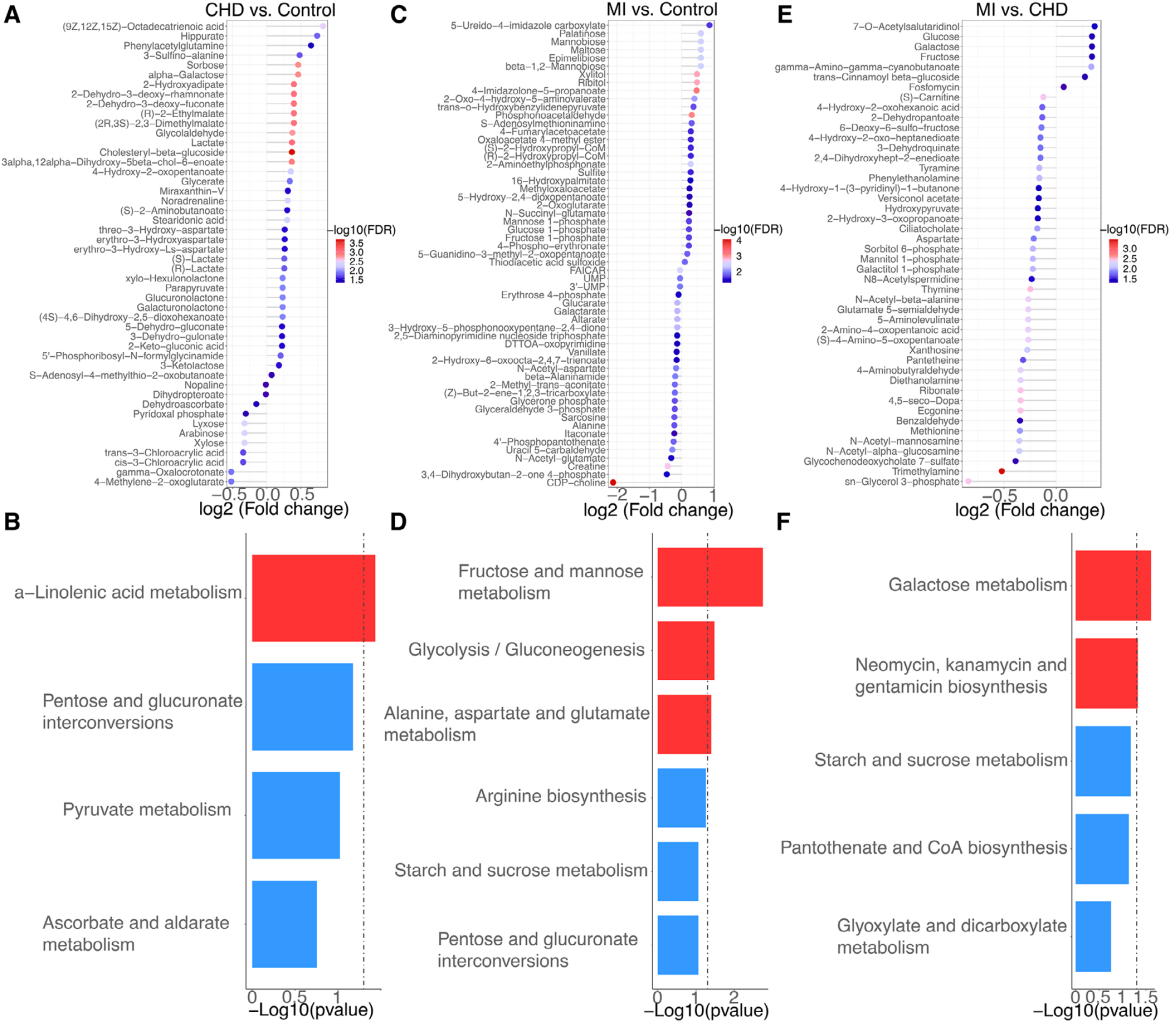
Metabolic profiles of MI, CHD and Control. (A) 48 metabolites exhibited specific differentiation between CHD (*n* = 83) and Control (*n* = 87). (B) KEGG pathway enrichment analysis using 48 metabolites (Hypergeometric test). (C) 57 metabolites exhibited specific differentiation between MI (*n* = 73) and Control (*n* = 87). (D) KEGG pathway enrichment analysis using 57 metabolites (Hypergeometric test). (E) 46 metabolites exhibited specific differentiation between CHD (*n* = 83) and MI (*n* = 73). (F) KEGG pathway enrichment analysis using 46 metabolites (Hypergeometric test). *p* Values were calculated by Wilcoxon test.

It is a clinical challenge to predict whether or when patients with CHD will develop MI. Here we found significant metabolic alterations between CHD and MI in PCA and PLS‐DA analyses, in which 46 metabolites were specifically dysregulated (Figure 2C) with 39 downregulated and 7 upregulated metabolites (Figure 3E), respectively. KEGG pathway analysis found two dysregulated pathways: galactose metabolism and neomycin, kanamycin and gentamicin biosynthesis pathway (Figure 3F).

Following comprehensive metabolic profiling, we performed biomarker discovery analysis to predict CHD and MI and differentiate individual patients based on their serum metabolomics data. In this direction, we established a LASSO regression model to select a panel of metabolites as potential biomarkers by randomly grouping 2/3 of the samples into a training set and the rest of samples as a test set.^8^ To increase the confidence and reproducibility of analysis, we only chose the metabolites with molecular ions of [M+H]^+^ and [M‐H]^−^ in MS^1^ scan and Grade 1 classification based on Metabolomics Standards Initiative (MSI) criteria.^9^ We subsequently performed a receiver operator characteristic (ROC) curve analysis on the training and test datasets.^10^ We identified three metabolites, acetylcarnitine, arginine and hypoxanthine, as potential biomarkers in the LASSO model to differentiate MI from CHD (Figure 4A). The importance of these putative biomarkers was shown in Figure 4B. The AUC was .92 and .88 in the training and test set, respectively (Figure 4C), and the relative abundance of putative biomarkers was illustrated using a boxplot (Figure 4D). Four metabolites, sphingomyelin, citrulline, glutamate and hexadecenoic acid, were selected to differentiate CHD from control groups with an AUC of .82 and .79 in the training and test set, respectively (Figure S1A). Glutamate was the most important metabolite in our statistic model (Figure S1B and C). Interestingly, these four metabolites were all upregulated in the CHD group (Figure S1D). Lastly, four metabolites, acetylcarnitine, 5‐methylthioaenosine, salicyluric acid and hypoxanthine, were used to distinguish MI patients from the control (Figure S2A). The AUC was .95 and .94 in the training and test set, respectively (Figure S2B and C). 5‐Methylthioaenosine was the most important metabolite in this prediction model (Figure S2B) and was the only downregulated metabolite among these putative biomarkers (Figure S2D). Finally, we structurally validated all these putative biomarkers using commercial standards (Figure S3). Notably, we attempted to incorporate conventional clinical indices, such as LDL‐C, ApoB and apoA1, into our predictive models but did not observe significant improvements (data not shown).

**FIGURE 4:**
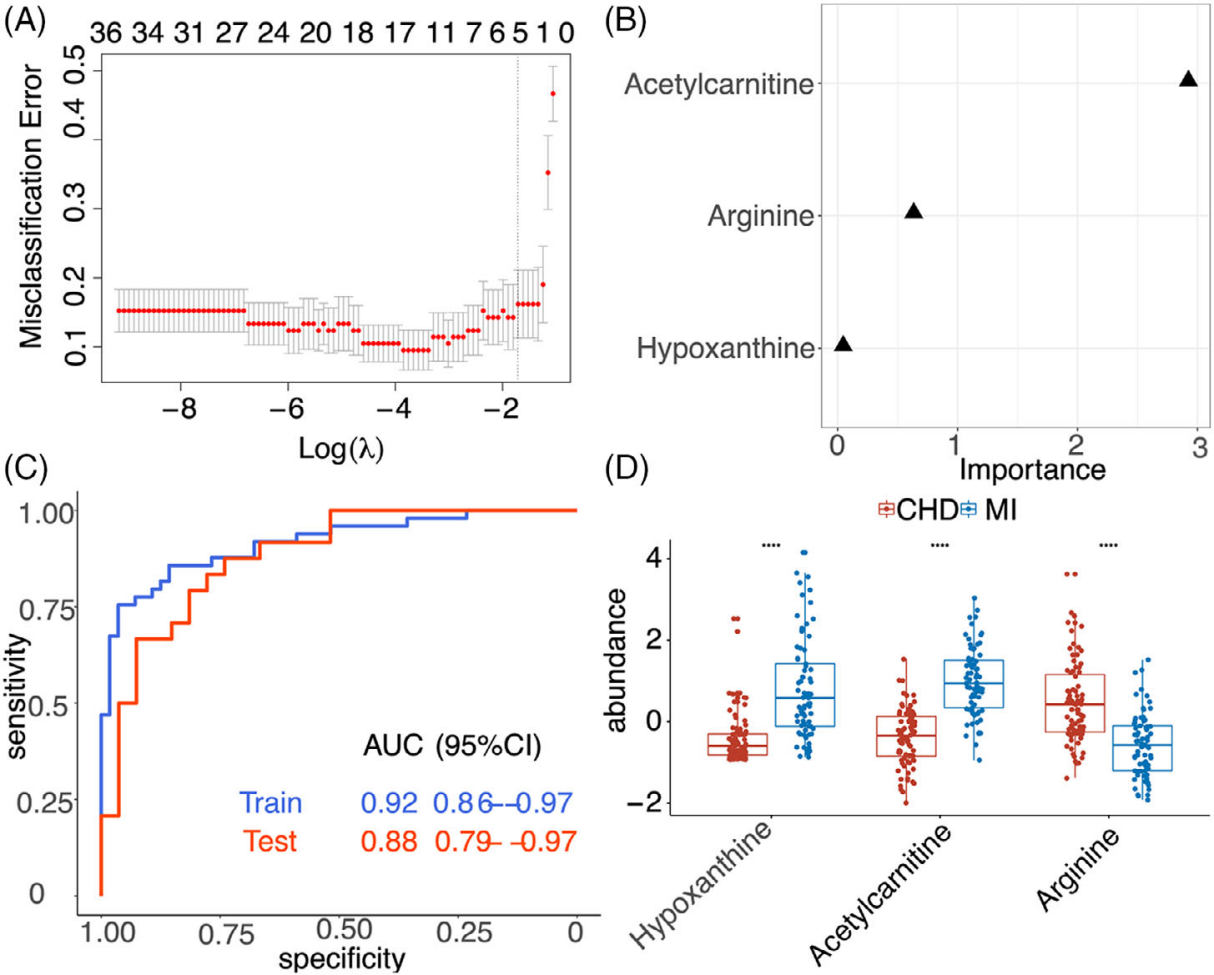
Biomarker discovery between CHD and MI. (A) Variable selection‐based LASSO algorithm. (B) The variable importance of selected biomarkers. (C) Receiver operator characteristics (ROC) analysis of prediction model. (D) The box plot of biomarkers in CHD (*n* = 83) and MI (*n* = 73). Data in (D) were presented as box plots (median and IQR) with single data points superimposed. *p* Values were calculated by Wilcoxon test.

In conclusion, the prediction models utilising serum metabolites selected from metabolomics and machine learning offers great potential in improving diagnosis of CHD and MI after validated in multicentred cohort studies.

## AUTHOR CONTRIBUTIONS

Xia Shen, Dewen Yan, Lemin Zheng and Huiyong Yin conceived the project and wrote the manuscript. Xia Shen, Huiyong Yin and Ningning Liang designed, performed experiments and analysed the data. Shuyuan Guo, Zi Li and Ningning Liang helped with LCMS experiments. Mingming Zhao and Chun Wang helped with sample collection and provided patient information. Xia Shen and Huiyong Yin edited the manuscript. Huiyong Yin and Lemin Zheng supervised the work.

## FUNDING INFORMATION

This research was funded by grants from the National Key Research and Development Program of China (2022YFC2503300) and National Natural Science Foundation of China (32241017 and 32030053), Shenzhen Medical Research Fund (SMRF B2302042), Science and Technology Commission of Shanghai Municipality (22140903300), and startup funds from the City University of Hong Kong (9380154 and 7006046), RGC Theme‐based Research Scheme (8770011), and TBSC Project fund. DWY was supported by Shenzhen Clinical Research Center for Metabolic Diseases (Shenzhen Science, Technology and Innovation [2021]287), Shenzhen Center for Diabetes Control and Prevention (NO: SZMHC [2020]46).

## CONFLICT OF INTEREST STATEMENT

None declared.

## ETHICS STATEMENT

This study was approved by the Ethics Committee of the Fujian Provincial Hospital, which is in accordance with the Declaration of Helsinki. Informed consents were obtained from all participants.

## Supporting information

Supporting Information

## Data Availability

The data supporting the findings of this study are available from the corresponding author upon reasonable request.

## References

[1] Karjalainen MK , Karthikeyan S , Oliver‐Williams C , et al. Genome‐wide characterization of circulating metabolic biomarkers. Nature. 2024;628(8006):130‐138. 38448586 10.1038/s41586-024-07148-y PMC10990933

[2] Lu J , Chen B , Chen T , et al. Comprehensive metabolomics identified lipid peroxidation as a prominent feature in human plasma of patients with coronary heart diseases. Redox Biol. 2017;12:899‐907. 28472752 10.1016/j.redox.2017.04.032 PMC5415551

[3] Cavus E , Karakas M , Ojeda FM , et al. Association of circulating metabolites with risk of coronary heart disease in a European Population: results from the Biomarkers for Cardiovascular Risk Assessment in Europe (BiomarCaRE) Consortium. JAMA Cardiol. 2019;4(12):1270‐1279. 31664431 10.1001/jamacardio.2019.4130 PMC6822093

[4] Wittenbecher C , Eichelmann F , Toledo E , et al. Lipid profiles and heart failure risk: results from two prospective studies. Circ Res. 2021;128(3):309‐320. 33272114 10.1161/CIRCRESAHA.120.317883 PMC7864881

[5] Shen X , Liang N , Liu Z , et al. Serum metabolomics identifies dysregulated pathways and potential metabolic biomarkers for hyperuricemia and gout. Arthritis Rheumatol. 2021;73(9):1738‐1748. 33760368 10.1002/art.41733

[6] Shao F , Li R , Guo Q , et al. Plasma metabolomics reveals systemic metabolic alterations of subclinical and clinical hypothyroidism. J Clin Endocrinol Metab. 2022;108(1):13‐25. 36181451 10.1210/clinem/dgac555 PMC9759175

[7] Triba MN , Le Moyec L , et al. PLS/OPLS models in metabolomics: the impact of permutation of dataset rows on the K‐fold cross‐validation quality parameters. Mol Biosyst. 2015;11(1):13‐19. 25382277 10.1039/c4mb00414k

[8] Tibshirani R . The lasso method for variable selection in the Cox model. Stat Med. 1997;16(4):385‐395. 9044528 10.1002/(sici)1097-0258(19970228)16:4<385::aid-sim380>3.0.co;2-3

[9] Sumner LW , Amberg A , Barrett D , et al. Proposed minimum reporting standards for chemical analysis Chemical Analysis Working Group (CAWG) Metabolomics Standards Initiative (MSI). Metabolomics. 2007;3(3):211‐221. 24039616 10.1007/s11306-007-0082-2 PMC3772505

[10] Nettleman MD . Receiver operator characteristic (ROC) curves. Infect Control Hosp Epidemiol. 1988;9(8):374‐377. 3049782 10.1086/645891

