# Supplementary material for: Biomarker discovery and metabolic profiling in serum of cardiovascular disease patients with untargeted metabolomics and machine learning
Source: Clin Transl Med. 2024 Jun 20;14(6):e1722. doi: 10.1002/ctm2.1722 (PMC11187800; doi:10.1002/ctm2.1722)
Supplement: Supplementary file 1 — Supporting Information [file CTM2-14-e1722-s001.docx]

**Supplementary Materials**

**1. Detailed method of metabolic profiling analysis of serum samples**

The serum sample preparation and LC-MS methods were listed in previous publications[1]. The raw MS data was analyzed followed by the standard procedure according to previous publication[2]. The SERRF normalization method was used to normalize the original data[3]. Peaks with RSD (relative standard deviation) higher than 30% in QC samples were removed from the peak table. MetDNA webserver was used for the metabolite identification[2].

**2. Methods**

**2.1 Subjects**

A total of 243 participants (73 MI patients, 83 CHD patients, and 87 control control) were enrolled at the Fujian Provincial Hospital, China. This study was approved by the Human Ethics Committee of Fujian Provincial Hospital. Detailed clinical characteristics for these participants were shown in Table 1. The enrollment criteria of the study subjects are set as follows: all selected patients have not received other treatment before admission; all subjects have no related metabolic diseases, such as kidney disease, liver disease, and other types of tumors. The control group refers to the coronary blood vessels without stenosis, but the blood lipids are abnormal; the coronary heart disease group refers to the intravascular stenosis >50%, and the myocardial infarction group refers to the patients with typical three-vessel disease, which causes 100% blood vessel blockage. The venous blood samples were taken without any medication after overnight fasting. The blood was allowed to clot for 30 min at room temperature before taken the supernatant after centrifuged at 3000 rpm for 10 min. The serum samples were aliquoted and stored immediately at -80 ºC till sample preparation and LC-MS analysis**.** Pooled samples were used as QC.

**2.2 Statistical analysis**

The study design and data analysis workflow were illustrated in Figure 1. We performed univariate analysis with Wilcoxon tests to explore the statistical significance by comparisons CHD with Control, MI with Control and MI with CHD group.

Unsupervised multi-variate analysis, principal component analysis (PCA, *R package MixOmics*) and supervised Partial least-squares Discriminate Analysis (PLS-DA, *R package MixOmics*) were used to maximize the global metabolic variations among groups. Hierarchical cluster analysis (R *package Complexheatmap*) was utilized to explore the metabolites changing trend among three groups. Pathway enrichment analysis was conducted by MetaboAnalyst website. The metabolites which FDR < 0.05 were mapped into KEGG database with the Hypergeometric Test to calculate significant perturbed pathways.

In biomarker discovery, we used LASSO (Least absolute shrinkage and selection operator) to select potential biomarkers. To control the simplicity of the predictive model, we increase the lambda values to limit the numbers of variables less than 5 [4]. Subsequently, the prediction models were established in training and testing datasets and ROC analysis used to evaluate the specificity and sensitivity of our model. Notably, there is no overlap in training and test data set. The ROC (receiver operating characteristic) conducted by R package pROC was applied to evaluate the performance of our model. The bootstrap method used to calculate the AUC in different data split for 1001 times, the median value of AUC was chosen as the final statistical model. All statistical analyses were performed using R software (version 3.6.1).

**Figure S1**

**Biomarker discovery between CHD and Control.** (A) Variable selection-based LASSO algorithm. (B) The variable importance of selected biomarkers. (C) Receiver operator characteristics (ROC) analysis of prediction model. (D) The box plot of biomarkers in CHD (n = 83) and Control (n = 87). Data in (D) was presented as box plots (median and IQR) with single data points superimposed. *P* values were calculated by Wilcoxon test.

**Figure S2**

**Biomarker discovery between MI and Control.** (A) Variable selection-based LASSO algorithm. (B) The variable importance of selected biomarkers. (C) Receiver operator characteristics (ROC) analysis of prediction model. (D) The box plot of biomarkers in MI (n = 73) and Control (n = 87). Data in (D) was presented as box plots (median and IQR) with single data points superimposed. *P* values were calculated by Wilcoxon test.

**
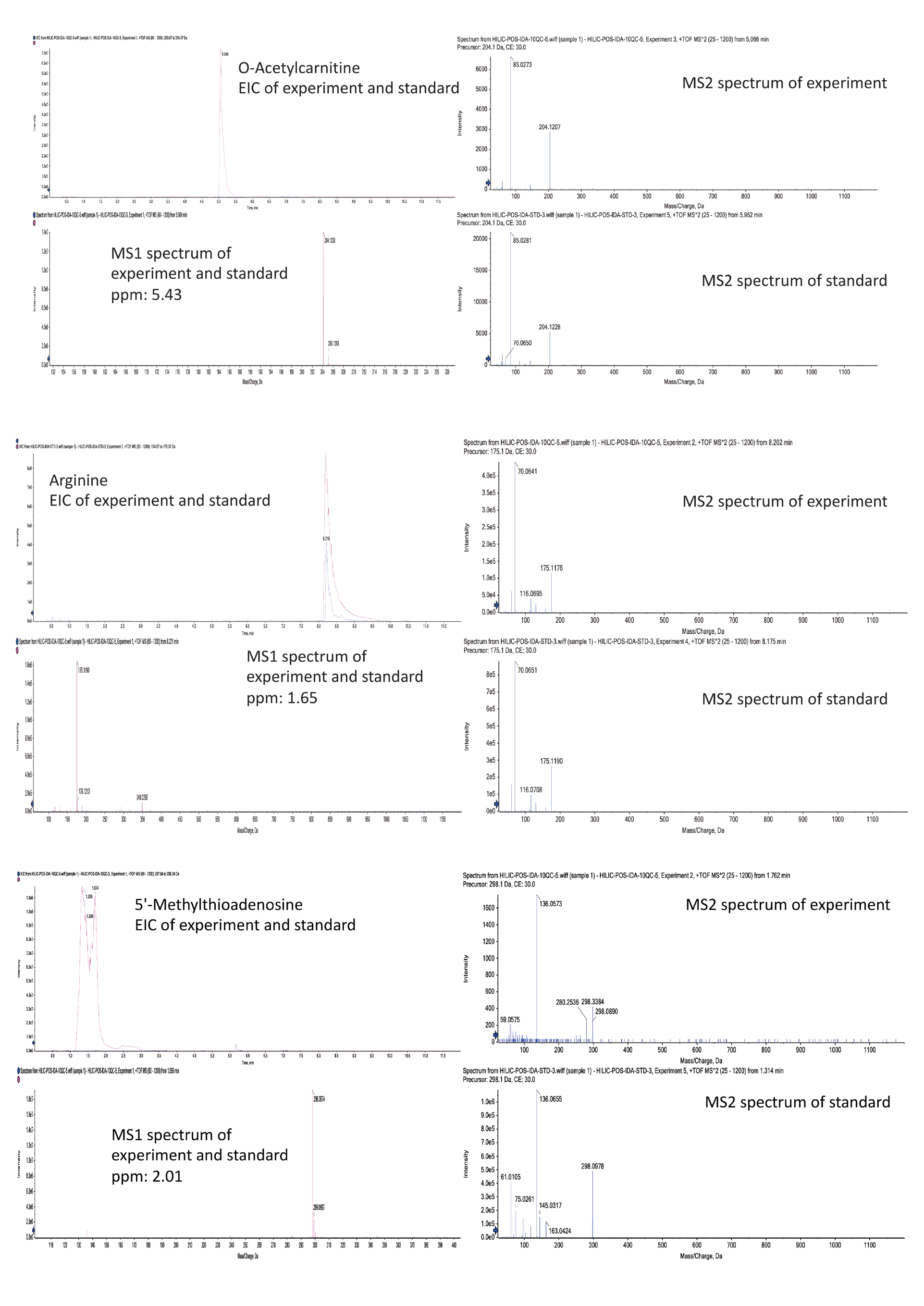
**

**
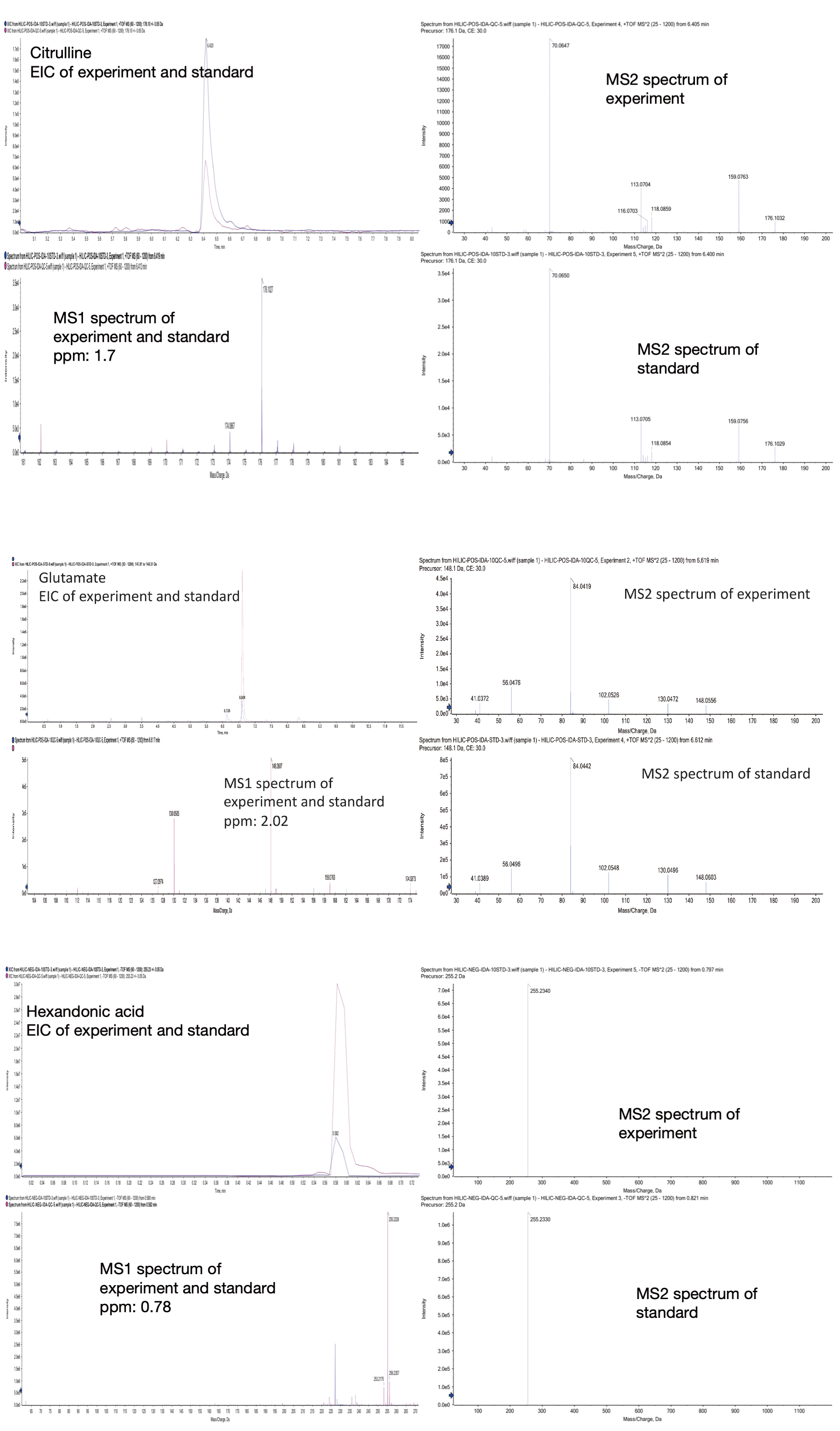
**

**
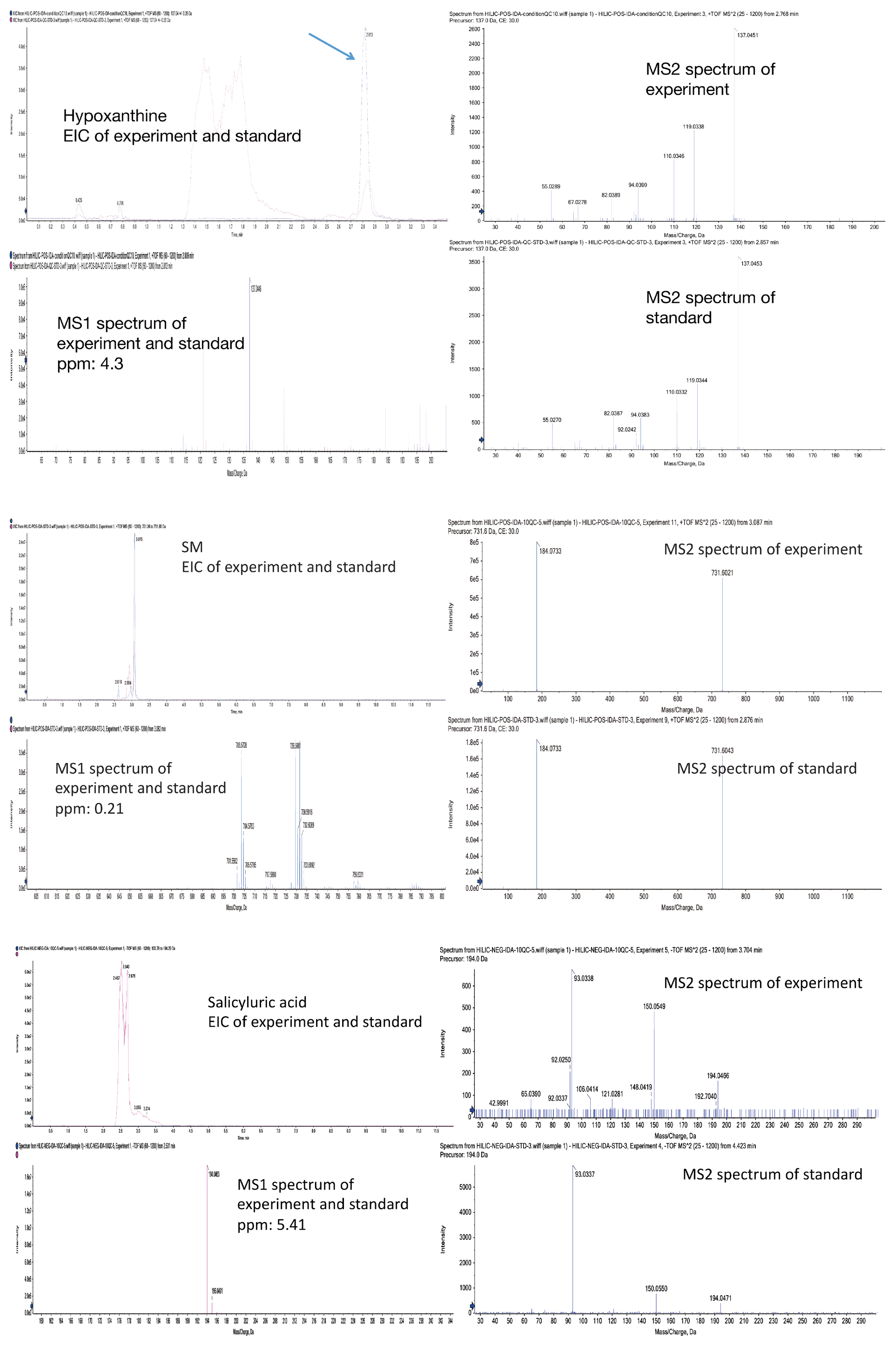
**

**Figure S3**

The MS1 and MS2 information of biomarkers.

**Reference：**

1. Shen, X., et al., *Serum Metabolomics Identifies Dysregulated Pathways and Potential Metabolic Biomarkers for Hyperuricemia and Gout.* Arthritis Rheumatol, 2021. **73**(9): p. 1738-1748.

2. Shen, X., et al., *Metabolic reaction network-based recursive metabolite annotation for untargeted metabolomics.* Nat Commun, 2019. **10**(1): p. 1516.

3. Fan, S., et al., *Systematic Error Removal Using Random Forest for Normalizing Large-Scale Untargeted Lipidomics Data.* Anal Chem, 2019. **91**(5): p. 3590-3596.

4. Liang, L., et al., *Metabolic Dynamics and Prediction of Gestational Age and Time to Delivery in Pregnant Women.* Cell, 2020. **181**(7): p. 1680-1692 e15.
